# A necroptosis‐related gene signature for predicting prognosis, immune landscape, and drug sensitivity in hepatocellular carcinoma

**DOI:** 10.1002/cam4.4812

**Published:** 2022-05-13

**Authors:** Junliang Chen, Huaitao Wang, Lei Zhou, Zhihao Liu, Hui Chen, Xiaodong Tan

**Affiliations:** ^1^ Department of General Surgery Shengjing Hospital of China Medical University Shenyang Liaoning P. R. China

**Keywords:** gene signature, hepatocellular carcinoma, necroptosis, nomogram, prognosis, therapy

## Abstract

**Background:**

Hepatocellular carcinoma (HCC) remains a growing threat to global health. Necroptosis is a newly discovered form of cell necrosis that plays a vital role in cancer development. Thus, we conducted this study to identify a predictive signature of HCC based on necroptosis‐related genes.

**Methods:**

The tumor samples in the liver hepatocellular carcinoma (LIHC) cohort from The Cancer Genome Atlas (TCGA) database were subtyped using the consensus clustering algorithm. Univariate Cox regression and LASSO‐Cox analysis were performed to identify a gene signature from genes differentially expressed between tumor clusters. Then, we integrated the TNM stage and the prognostic model to build a nomogram. The gene signature and the nomogram were externally validated in the GSE14520 cohort from the Gene Expression Omnibus (GEO) and the LIRP‐JP cohort from the International Cancer Genome Consortium (ICGC). Evaluations of predictive performance evaluations were conducted using Kaplan–Meier plots, time‐dependent receiver operating characteristic curves, principal component analyses, concordance indices, and decision curve analyses. The tumor microenvironment was estimated using eight published methods. Finally, we forecasted the sensitivity of HCC patients to immunotherapy and chemotherapy based on this gene signature.

**Results:**

We identified two necroptosis‐related clusters and a 10‐gene signature (MTMR2, CDCA8, S100A9, ANXA10, G6PD, SLC1A5, SLC2A1, SPP1, PLOD2, and MMP1). The gene signature and the nomogram had good predictive ability in the TCGA, ICGC, and GEO cohorts. The risk score was positively associated with the levels of necroptosis and immune cell infiltrations (especially of immunosuppressive cells). The high‐risk group could benefit more from immunotherapy and some chemotherapeutics than the low‐risk group.

**Conclusion:**

The necroptosis‐related gene signature provides a new method for the risk stratification and treatment optimization of HCC. The nomogram can further improve predictive accuracy.

## Graphical abstract

Necroptosis is a newly discovered form of cell necrosis that plays a vital role in cancer development. We identified a necroptosis‐related gene signature based on the TCGA cohort, which was validated externally in the ICGC‐LIRI‐JP and GSE14520 cohorts. Our study linked necroptosis with the prognosis, immune landscape, and drug sensitivity of HCC patients.

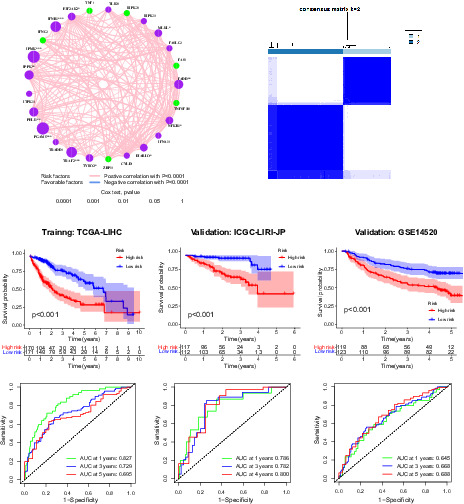

## 1 BACKGROUND

Hepatocellular carcinoma (HCC), the fourth most common cause of cancer mortality, is projected to result in the deaths of more than one million people in 2025 worldwide.^1^ Although substantial progress has been achieved in surveillance and systemic treatment of HCC, it is still the second deadliest cancer with a dismal overall five‐year survival rate of 18%.^2^ Due to the heterogeneity of HCC resulting from different etiological factors, prognostic prediction based on the TNM strategy alone is challenging.^3^ Furthermore, new therapeutic targets are urgently warranted to guide clinical practice and optimize medical interventions. Thus, a novel prognostic model utilizing molecular profiles of HCC is highly desirable.

Necroptosis is a newly discovered form of cell necrosis and is mainly mediated by necrosomes, which comprise mixed lineage kinase domain‐like protein (MLKL), receptor‐interacting protein kinases 1 (RIPK1), and RIPK3.^4^ Differing from apoptosis in morphology, necroptosis is characterized by cellular swelling and membrane perforation with subsequent release of pro‐inflammatory damage‐associated molecular patterns (DAMPs). When caspase is inhibited, necroptosis functions as a backup system to combat pathogens or cancer cells that evade apoptotic processes, which is also one of the vital mechanisms for some antineoplastic drugs, such as 5‐FU, etoposide, and cisplatin.^5^, ^6^, ^7^, ^8^ In addition to its defensive effect against malignancy, accumulating evidence suggests that necroptosis can promote tumorigenesis, cancer metastasis, and immunosuppression. For example, RIPK1 is overexpressed in glioblastoma and associated with poor prognosis by attenuating p53 activation.^9^ Knockout of RIPK1, RIPK3, and MLKL in colon and esophageal cancer cells can significantly suppress tumor growth by decreasing the activity of NF‐kappaB (NF‐κB).^10^ Taken together, these data suggest the role of necroptosis in cancer is highly complex and ambivalent. Additionally, the relationship between necroptosis and overall survival (OS) remains largely unknown in HCC patients.

In the present study, a prognostic model was established according to genes differentially expressed (DEGs) between two necroptosis‐related HCC subtypes. Next, the molecular signature was integrated with the TNM stage to build a nomogram for better predictive ability. Ultimately, we investigated the immune landscape of the tumor microenvironment (TME) and predicted sensitivities to pharmacotherapeutics. Our findings provide reliable evidence for the risk stratification and treatment optimization of HCC patients.

## 2 MATERIALS AND METHODS

### 2.1 Dataset acquisition

The process used for analysis is summarized in the flow chart (Figure S1). Data from 371 HCC samples in the liver hepatocellular carcinoma (LIHC) cohort were downloaded from The Cancer Genome Atlas (TCGA) repository, including fragments per kilobase of transcript per million mapped reads (FPKM) normalized values, corresponding clinical information, and single nucleotide variation (SNV) and copy number variation (CNV) data (https://portal.gdc.cancer.gov/repository). Only 341 patients with enough follow‐up time (≥30 days) were subjected to prognostic model fitting. For external validation of this model, the RMA‐normalized matrix from the GSE14520 cohort (including 242 tumor samples) was downloaded from the Gene Expression Omnibus (GEO), and raw count data from the LIRI‐JP cohort (including 231 tumor samples) were acquired from the International Cancer Genome Consortium (ICGC) (https://www.ncbi.nlm.nih.gov/geo/, https://dcc.icgc.org/projects/LIRI‐JP). Next, the mRNA sequencing data of the TCGA‐LIHC and ICGC‐LIRI‐JP cohorts were converted into transcripts per kilobase million (TPM) values, and then log2(*x* + 1) transformed, which was suggested to be the most accurate quantification method with minimal statistical biases.^11^, ^12^ The batch effects of the above three datasets were eliminated by the “Combat” function of the “sva” package. Due to a limited sample size of only 50 matched tumor‐normal samples in the TCGA‐LIHC cohort, another four datasets were included and selected by least absolute shrinkage and selection operator (LASSO)‐penalized Cox regression to validate the gene transcription levels, including GSE14520 (213 pairs), GSE25097 (243 pairs), GSE57957 (37 pairs), and GSE76297 (58 pairs).

### 2.2 Investigation of the expression levels of necroptosis‐related genes

Data regarding 25 key regulators of necroptosis were extracted from previous reviews and MSigDB (http://www.broadinstitute.org/gsea/msigdb/, M24779), which are presented in Table S1.^13^, ^14^, ^15^, ^16^, ^17^ SNV and CNV analyses were performed utilizing the “maftools” and “Rcircos” packages, respectively.^18^, ^19^ A network was plotted by the “igraph” package to summarize the expression correlations among necroptosis‐related genes and their prognostic values in the TCGA cohort.

### 2.3 Consensus clustering

Consensus clustering is a robust unsupervised classification technique achieved through multiple resampling and clustering. Regulators of necroptosis with prognostic values were used to classify patients. Similarity within each group was measured by Euclidean distance, and the whole process was repeated 1000 times using the “ConsensuClusterPlus” package.^20^ The appropriate cluster number (*k*) was determined according to the cumulative distribution function (CDF) plot. The flatness of the CDF curve indicates the level of consensus and the stability of clustering, which was further verified in principal component analysis (PCA).

### 2.4 Identification of DEGs between two necroptosis‐related clusters

DEGs between two necroptosis‐related clusters were identified by the “limma” package.^21^ A false discovery rate (FDR) < 0.05 and |log2FC| > 1 were criteria for statistical significance. The biological processes were assessed using Gene Ontology (GO) enrichment analysis based on the Metascape website (http://metascape.org). Based on the reference gene set obtained from MSigDB (c2.cp.kegg.v7.4.symbols), we converted the DEG expression data into a pathway score matrix using the “GSVA” package.^22^ The pathway activities were also compared by the “limma” package.

### 2.5 Development and validation of the necroptosis‐related prognostic model

Univariate Cox analysis was carried out to determine the DEGs associated with overall survival. Next, LASSO‐penalized Cox regression was employed to filter features to establish a risk model by the “glmnet” package.^23^, ^24^ The following formula was used to calculate the risk score for each patient: $\sum_{i=1}^{n}\text{expression}i*\text{coefficient}\;i$. According to the median risk score, patients in the training cohorts were classified into high‐ or low‐risk groups. Similarly, the risk scores of external validation cohorts (ICGC and GEO) were calculated using the same formula developed from the training group and divided based on the median value of the TCGA cohort.

### 2.6 Establishment and validation of the nomogram

We combined univariate and multivariate Cox analyses to determine the independent prognostic factors, which were subsequently incorporated to fit a Cox proportional hazard model.^25^ For convenience and clinical utility, a nomogram was established to visualize the results of the Cox model. Next, we carried out external validations in the ICGC and GEO cohorts to confirm the reliability of the nomogram. The total points of patients in the validation cohorts were calculated according to the Cox model derived from the TCGA cohort. Then Cox regressions were implemented again using the total points as a factor (nomogram). In turn, the concordance indices (C indices) and calibration curves were yielded based on the Cox results.^26^ In addition, we also plotted the time‐dependent receiver operating characteristic (ROC) curve and performed decision curve analysis (DCA).

### 2.7 Determination of the immune landscape

To decipher the immune cell infiltration, we employed single‐sample gene set enrichment analysis (ssGSEA)^22^ and seven deconvolution algorithms to fully leverage the transcription data, including the ESTIMATE,^27^ CIBERSORT,^28^ MCPCounter,^29^ xCell,^30^ EPIC,^31^ quanTIseq,^32^ and TIMER.^33^ The gene set used by ssGSEA was obtained from Charoentong's study.^34^ The whole process of TME estimation was carried out by the “IOBR” package integrating the above eight methodologies.^35^

### 2.8 Prediction of sensitivity to chemotherapy and immunotherapy

Aiming to improve personalized treatment, we employed the “oncoPredict” package to build ridge regression models to estimate the half‐maximal effective concentration (EC50) values, which were then compared between high‐ and low‐risk groups.^36^ The data of cell lines in the Cancer Therapeutics Response Portal (CTRP) V2.1 were used as the training cohort (https://portals.broadinstitute.org/ctrp.v2.1). A lower EC50 value indicates a higher drug efficacy. Then, GSEA analyses were carried out to explore the differences in pathway activities between the two groups using GSEA software (version 4.22).

To investigate immunotherapy, we first uploaded the normalized sequencing data of TCGA to the Tumor Immune Dysfunction and Exclusion (TIDE) website (http://tide.dfci.harvard.edu/), a computational prediction tool based on the expression profiles of pretreated tumors.^37^ Due to the lack of published data on immune checkpoint inhibitors (ICIs) for HCC, we validated the gene signature in the IMvigor210 cohort comprising patients with metastatic urothelial cancers.^38^ The risk score distributions of the complete/partial response (CR/PR) and stable/progressive disease (SD/PD) group data were further explored.

### 2.9 Statistical analysis

All the data analyses and visualizations were accomplished in R software (version 4.12). The Wilcoxon test was used to compare the differences between the two groups. Categorical data were compared using the chi‐square test. The correlation coefficient was determined by the nonparametric Spearman approach. PCA was completed by the “prcomp” function of the “stats” package. Kaplan–Meier (K‐M) analysis, log‐rank test, and Cox regression were performed using the “survival” and the “survminer” packages. The C index was calculated for each Cox model and pooled using the random‐effects mixed model with maximum likelihood.^39^ A time‐dependent ROC curve was created by the “timeROC” package to evaluate predictive ability. The “rms” package was employed to generate the nomogram and calibrate the plot. Two‐tailed *p* < 0.05 was considered significant.

## 3 RESULTS

### 3.1 Overview of the genetic and transcriptional variations of necroptosis‐related genes in HCC

**FIGURE 1:**
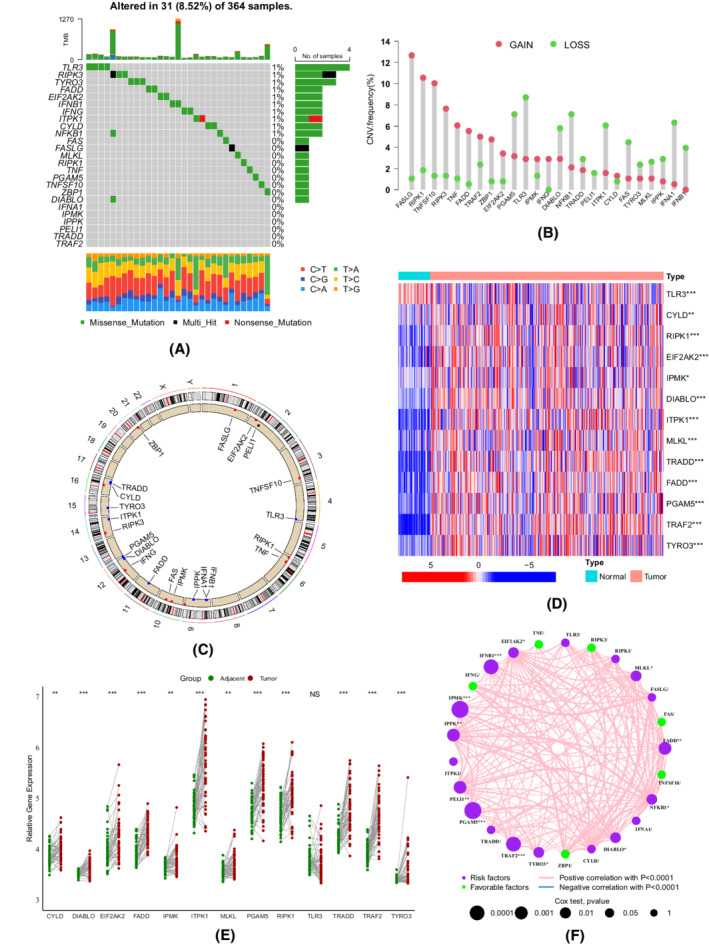
Genetic and transcriptional variations in necroptosis‐related genes in the TCGA‐LIHC cohort. (A) Oncoplot depicting the mutations of necroptosis‐related genes. (B) Copy number variation (CNV) frequencies of necroptosis‐related genes. (C) The locations of necroptosis‐related genes with CNV alterations on chromosomes. (D) The heatmap of differentially expressed necroptosis‐related genes between tumor and normal samples (Wilcox test, *p < 0.05; **p < 0.01; ***p < 0.001; ****p < 0.0001). (E) The mRNA levels of 13 necroptosis‐related genes in 50 matched tumor‐normal samples (Wilcox test). (F) A network plot illustrating the correlations among necroptosis‐related genes and their prognostic values

We first performed SNV and CNV analyses based on data from the TCGA‐LIHC cohort. At the genetic level, 31 of 364 patients had mutations in necroptosis‐related genes. Mutations in TLR3, RIPK3, and TYRO3 showed the highest frequencies (1%), while six genes (IFNA1, IPMK, IPPK, PELI1, TRADD, and TRAF2) did not display any alterations (Figure 1A). As illustrated in Figure 1B,C, genomic instabilities were widespread in necroptosis‐related genes, including 12 CNV gains, 12 CNV losses, and one nonsignificant alteration. The comparisons between 50 normal and 371 HCC samples indicated that 13 genes were significantly differentially expressed, including the upregulation of the levels of 12 genes and the downregulation of the levels of one gene in tumors (Figure 1D). The same tendency could also be observed in 50 pairs of matched tumor‐normal samples (Figure 1E). Additionally, we found that the gains or losses of copy number were not always positively correlated with the expression levels of necroptosis‐related genes, such as MLKL and TLR3, suggesting that CNV was not the only factor regulating gene expression.^40^ Some other mechanisms also play vital roles in gene expression, including DNA methylation, transcription factor activity, m6A modification, long noncoding RNAs, and RNA binding proteins.^41^, ^42^, ^43^, ^44^ Figure 1F depicts the correlations of these genes and their prognostic significance. Collectively, our results indicated that the expression levels of necroptosis‐related genes were significantly different between HCC and normal samples, which could be potential contributing factors to tumorigenesis and heterogeneity.

### 3.2 Identification of necroptosis‐related clusters in HCC

**FIGURE 2:**
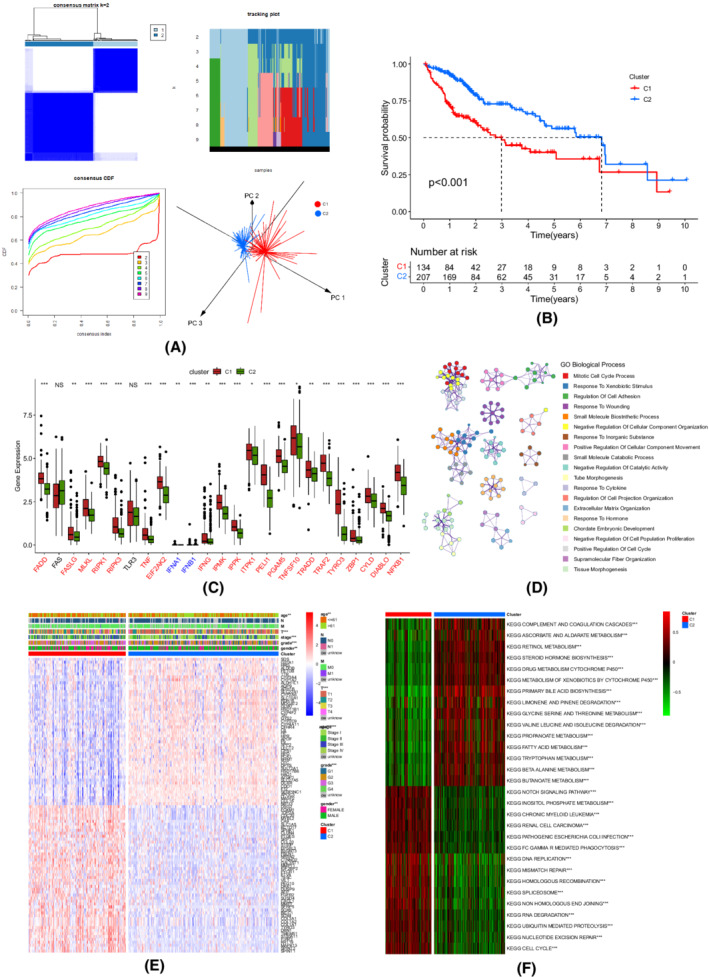
Identification of necroptosis‐related subgroups in the TCGA‐LIHC cohort. (A) Consensus matrix heatmap when *k* = 2; tracking plot depicting the assignment of items (in columns) at each *k* (in rows); cumulative distribution function (CDF) curve illustrating the stability at each *k*; principal component analysis (PCA) plot showing the differences between the two clusters. (B) Kaplan–Meier curve assessing the overall survival difference. (C) The expression levels of necroptosis‐related genes (Wilcox test, *p < 0.05; **p < 0.01; ***p < 0.001; ****p < 0.0001; NS, not statistically significant). (D) Biological process enrichment using the Metascape website. (E) Differences in clinicopathologic features and expression levels of necroptosis‐related genes (Bayes moderation). The top 100 of 985 DEGs were plotted in the heatmap. (F) Heatmap of GSVA analysis

We identified two molecular subtypes (134 patients in Cluster 1 and 207 patients in Cluster 2) according to the results of *k*‐means consensus clustering based on the 12 necroptosis‐related genes with prognostic value, and these subtypes were also validated by PCA (Figure 2A; Figure S2A–I; Table S2; Table S3). Cluster 1 patients had significantly worse overall survival (OS) (*p* < 0.001) and higher expression levels of most necroptosis‐related genes than those of Cluster 2 patients (Figure 2B,C). As presented in Table S4, there were significant differences between the patients in two clusters in transcription profile, age, T stage, TNM stage, tumor grade, and gender. The top 100 of the 985 DEGs were plotted in a heatmap (Figure 2E). Additionally, GSVA revealed that the pathways enriched in the C2 cluster were mainly associated with metabolism, biosynthesis, and degradation. Consistent with the results of GO enrichment, Cluster 1 showed enrichment for tumorigenesis, cell cycle, DNA replication, DNA repair, spliceosome, and immune activation pathways (Figure 2D,F; Table S5).

### 3.3 Development and validation of the 10‐gene signature based on DEGs between two necroptosis‐related clusters

Univariate Cox regression identified 376 DEGs related to overall survival in the TCGA‐LIHC cohort (*p* < 0.01; Table S6). Next, a 10‐fold cross‐validated Lasso‐Cox regression was performed to determine the coefficients for selected genes (Figure S3A,B). The risk scores of the TCGA, ICGC, and GEO cohorts were calculated as follows: risk score = (0.0276*MTMR2 expression) + (0.1250*CDCA8 expression) + (0.0291*SLC1A5 expression) + (0.0577*G6PD expression) + (0.0469*PLOD2 expression) + (0.0324*MMP1 expression) + (0.0385*SLC2A1 expression) − (0.0136*ANXA10 expression) + (0.0044*S100A9 expression) + (0.0050*SPP1 expression).

**FIGURE 3:**
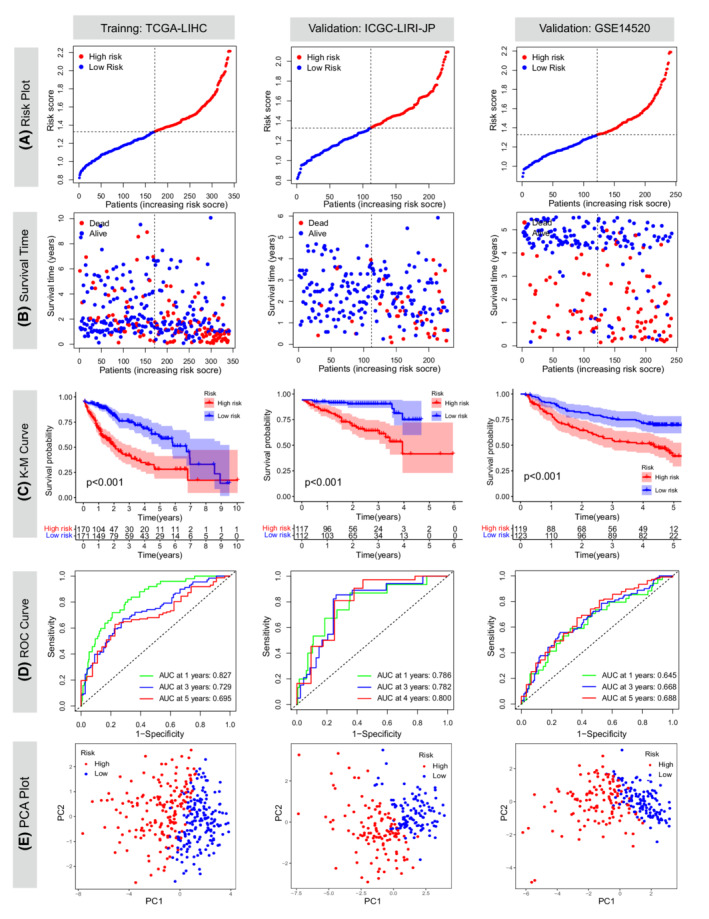
Prognostic values of the 10‐gene signature in TCGA‐LIHC, ICGC‐LIRI‐JP, and GSE14520 cohorts. (A) Median risk values and their distributions. (B) The correlations between risk scores and overall survival. (C) Kaplan–Meier curves assessing the differences in overall survival. (D) Time‐dependent receiver operating characteristic (ROC) curves and area under the curve (AUC) analyses. (E) Principal component analyses (PCAs)

**FIGURE 4:**
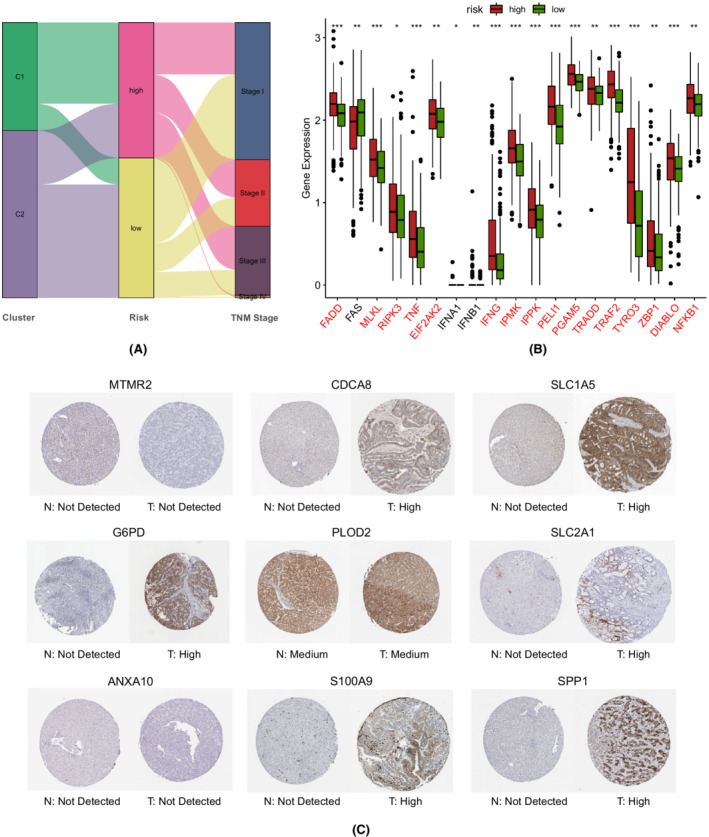
Association between the gene signature and necroptosis. (A) Sankey diagram depicting the associations between tumor clusters, risk groups, and TNM stages in the TCGA‐LIHC cohort. (B) The expression levels of necroptosis‐related genes between the high‐ and low‐risk groups (Wilcox test, *p < 0.05; **p < 0.01; ***p < 0.001; ****p < 0.0001). (C) Immunohistochemical images obtained from the Human Protein Atlas

According to the median risk value of the TCGA cohort, patients were split into low‐ and high‐risk groups (Figure 3A). The scatter plot indicated that risk scores were negatively associated with survival time (Figure 3B). Consistently, the K–M curves suggested that the prognosis of the high‐risk group was significantly worse than that of the low‐risk group (*p* < 0.001; Figure 3C). Subsequently, we performed time‐dependent ROC analyses to evaluate the predictive ability of this prognostic model. The areas under the curve (AUCs) were 0.827/0.729/0.695 at 1/3/5 years in the TCGA cohort, 0.786/0.782/0.800 at 1/3/4 years in the ICGC cohort, and 0.645/0.668/0.668 at 1/3/5 years in the GEO cohort (Figure 3D). PCA plots indicated that the high‐risk patients could be distinguished from their low‐risk counterparts (Figure 3E). Of note, the high‐risk group mainly comprised patients in the C1 cluster, which showed significantly higher expression levels of most necroptosis‐related genes, while the low‐risk group predominantly comprised patients in the C2 cluster (Figure 4A,B). The protein–protein interaction network between the 10 selected genes and necroptosis‐related genes is depicted in Figure S3D.

### 3.4 External validation of the expressions and genetic alterations of the 10 genes

**FIGURE 5:**
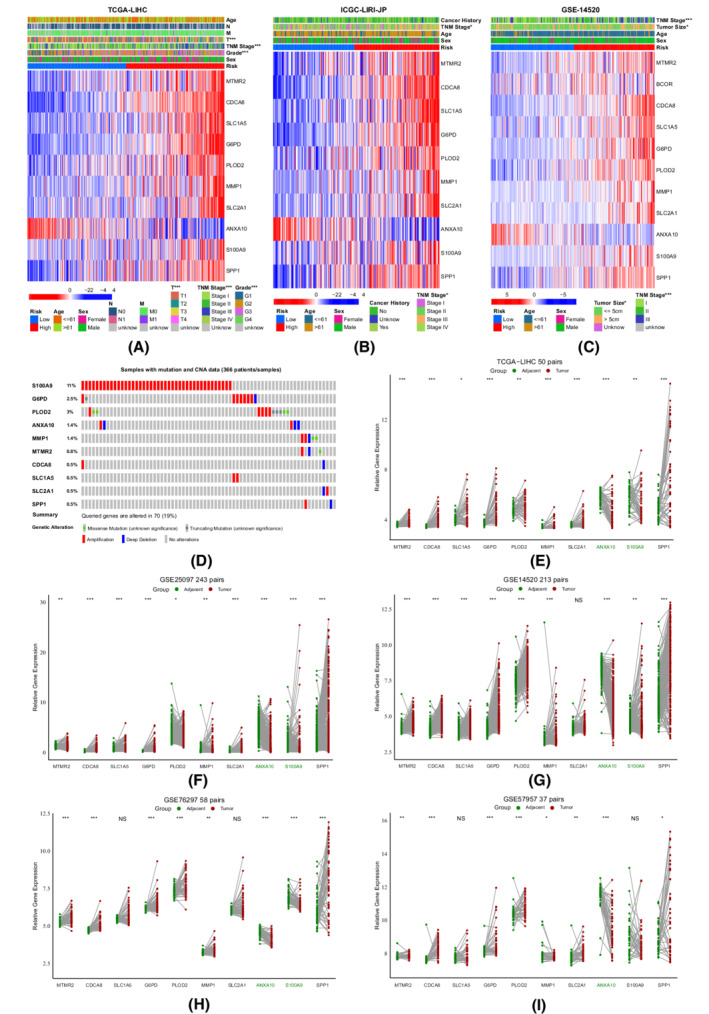
The expression and genetic alterations of the 10 selected genes. (A–C) The transcription levels of the 10 genes and clinical characteristics between the high‐ and low‐risk groups in the TCGA‐LIHC, ICGC‐LIRI‐JP, and GSE1450 cohorts. (D) Genetic alterations of the 10 genes in TCGA‐LIHC. (E–I) Validations of the expression levels of the 10 selected genes in the TCGA‐LIHC, GSE14520, GSE25097, GSE57957, and GSE76297cohorts

As no image for MMP1 immunohistochemical analysis was available from the Human Protein Atlas (https://www.proteinatlas.org/), typical images from the immunohistochemical analysis of the other nine genes were downloaded from this database (Figure 4C). We then investigated the transcription levels of the 10 genes in the TCGA, ICGC, and GEO cohorts, and found that risk scores were positively correlated with the expression levels of nine genes except for AXAN10 (Figure 5A–C). As depicted in the OncoPrint generated from cBioPortal (https://www.cbioportal.org), 70 out of 366 (19%) showed genetic alterations in the 10 genes, of which amplification was the most prevalent type (Figure 5D). Subsequently, five datasets with matched tumor‐normal samples were used to further validate the expression levels of the 10 genes (Figure 5E–I). Interestingly, S100A9 was paradoxically overexpressed in adjacent normal tissues, while the expression levels of the other nine genes were consistent with trends found between the high‐ and low‐risk groups. S100A9 protein, also known as myeloid‐related protein, typically resides in immune cells such as neutrophils, macrophages, and monocytes.^45^ Although S100A9 was overexpressed in liver cancer cells, it is also intensely upregulated in myeloid cells under pathological conditions, which may account for its higher expression levels in adjacent normal tissues.^46^ Therefore, we further compared the immune microenvironments between tumor and adjacent normal tissues, confirming that the fractions of neutrophils, M2 macrophages, and monocytes were indeed higher in adjacent normal tissues (Figure S4).

### 3.5 Establishment and validation of the predictive nomogram

**FIGURE 6:**
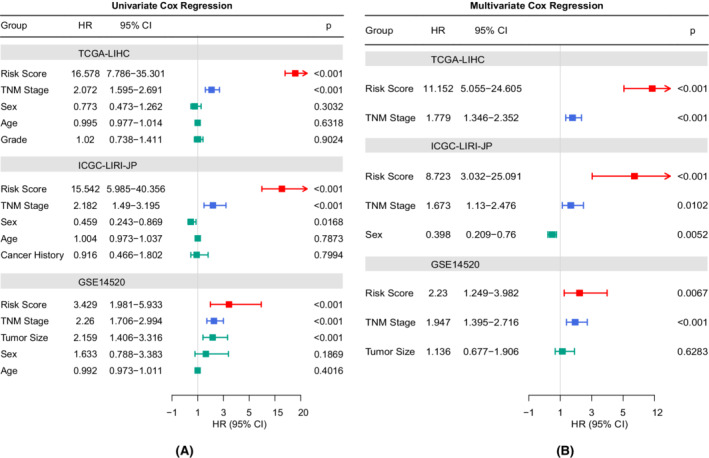
Identification of independent prognostic factors. (A) Results of the univariate Cox regression analyses. (B) Results of the multivariate Cox regression analyses

**FIGURE 7:**
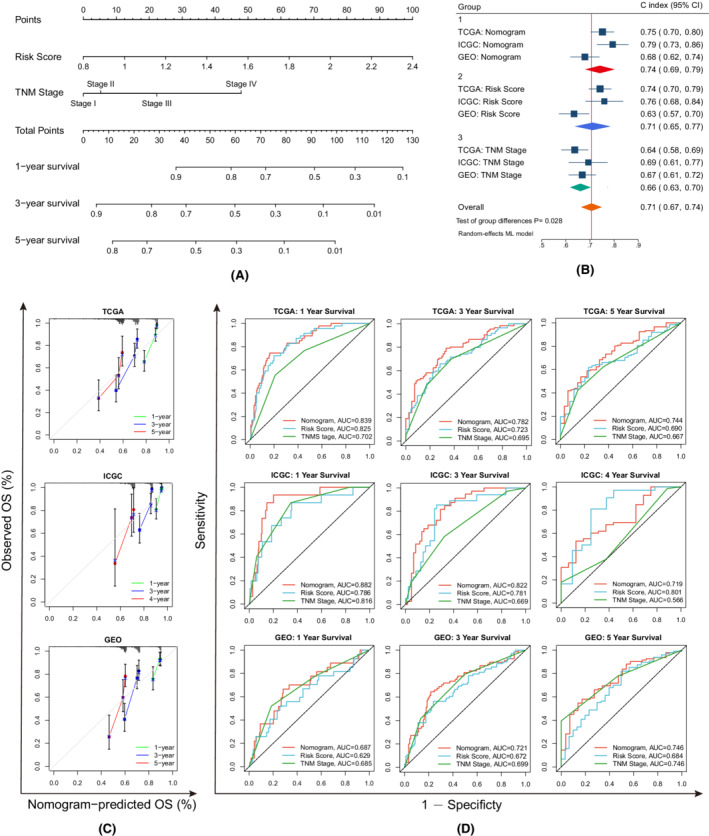
Development and validation of the predictive nomogram. (A) The nomogram is based on the TCGA‐LIHC cohort. (B) The forest plot depicting the pooled concordance indices of the nomogram, risk score, and TNM stage. (C) Calibration plots of the nomogram. (D) Time‐dependent ROC curves comparing the predictive capacities

Univariate and multivariate Cox analyses identified the risk score and TNM stage as independent prognostic factors in the TCGA, ICGC, and GEO cohorts, which were included to establish a nomogram (Figures 6A,B and 7A). The pooled C indices of the three cohorts demonstrated that the nomogram (0.74, 95% CI: 0.69–0.79) had better predictive performance than the risk score (0.71, 95% CI: 0.65–0.77) and TNM stage (0.66, 95% CI: 0.63–0.70) alone (Figure 7B). The calibration plots illustrated that the nomogram achieved good consistency between the predicted and observed OS outcomes (Figure 7C). The time‐dependent ROC curves showed that the nomogram had greater AUC values in most cases, further supporting its good discrimination ability (Figure 7D). According to the DCA analyses, the net benefits of the nomogram were evident for most patients (Figure S5).

### 3.6 Analyses of TME and drug sensitivity

**FIGURE 8:**
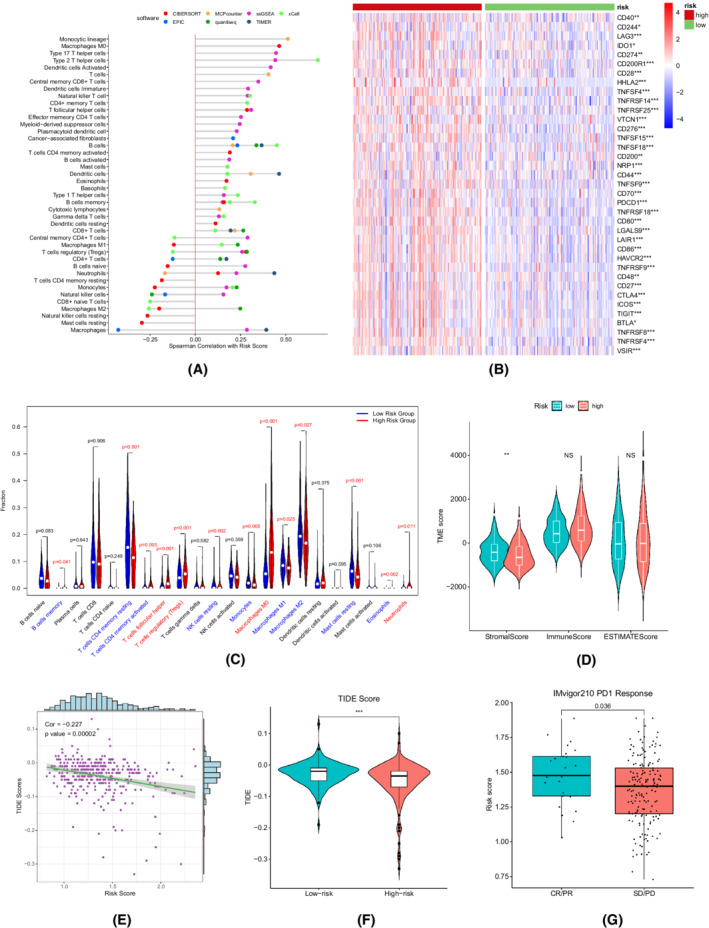
Exploration of the tumor microenvironment (TME) and response to immunotherapy in the TCGA‐LIHC cohort. (A) Correlations between the risk score and immune cell infiltration. (B) Comparisons of the expression of immune checkpoint‐related genes between the high‐ and low‐risk groups. (C) The estimated results of CIBERSORT. (D) Comparisons of the ESTIMATE scores between the high‐ and low‐risk groups. (E) Correlation between the Tumor Immune Dysfunction and Exclusion (TIDE) score and risk score. (F) Comparison of the TIDE scores between the high‐ and low‐risk groups (Wilcox test). (G) Comparison of the risk scores between the responders and nonresponders to anti‐PD1/PD‐L1 treatments in the IMvigor210 cohort

Given the importance of the risk score in prognosis prediction, we next investigated its potential value in reflecting the TME and guiding individualized treatment. The presence of most immune cells in the tumor microenvironment was positively correlated with the risk score, especially for cells facilitating HCC development and immunosuppression, including type 2 T helper (Th2) cells, type 17 T helper (Th17) cells, T regulatory (Treg) cells, myeloid‐derived suppressor cells (MDSCs), neutrophils, and cancer‐associated fibroblasts (CAFs) (Figure 8A,C).^47^, ^48^ M0 macrophages, B cells, dendrite cells, and T follicular helper cells exhibited the same trends. In contrast, the positive correlations between the risk score and the presence of tumor‐killing cells, such as gamma delta T cells (Tγδ), CD8+ T cells, and natural killer T (NKT) cells, were weaker. In addition, we found that the levels of 37 immune checkpoint‐related genes, such as PDCD1 (PD1), CD274 (PD‐L1), and CTLA4, were overexpressed in the high‐risk group, which further impaired antitumor immunity (Figure 8B). There was a tendency for high‐risk patients to have higher immune scores (Figure 8D). Subsequently, we utilized the TIDE algorithm to evaluate the responses of patients to immunotherapy. The risk score was negatively correlated with the TIDE score, and the high‐risk group had a significantly lower TIDE score, suggesting that the high‐risk group was potentially more sensitive to immunotherapy (Figure 8E,F). In addition, we found that responders (CR/PR) to anti‐PD1/PD‐L1 treatment had significantly higher risk scores in the IMvigor 210 cohort than those of nonresponders (Figure 8G).

**FIGURE 9:**
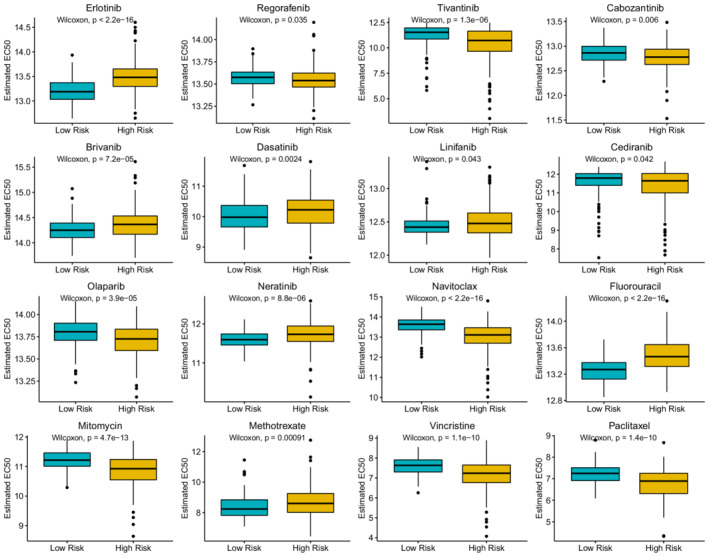
Drug sensitivity predictions based on the “oncoPredict” R package

**FIGURE 10:**
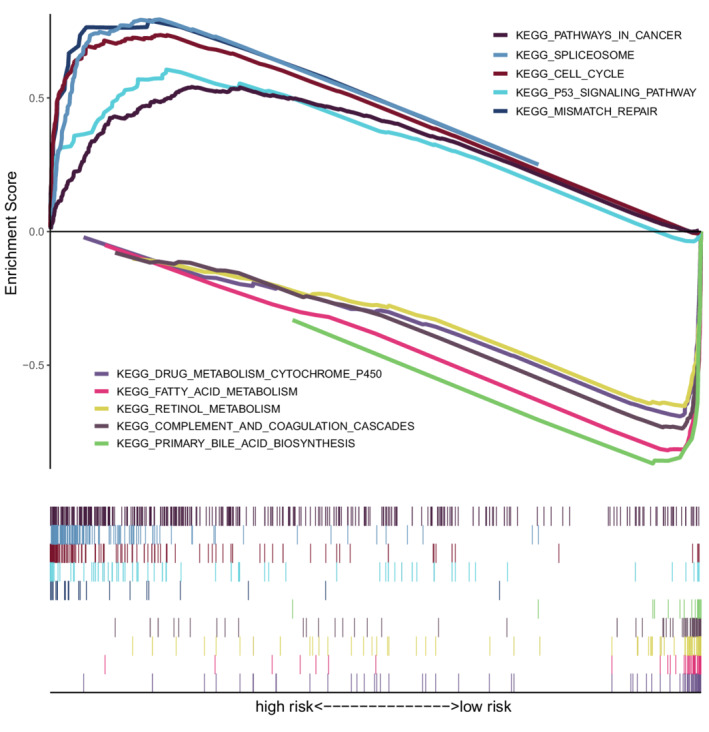
Results of the GSEA analyses

To better link the necroptosis‐related gene signature with clinical practice, we used the “oncoPredict” package to predict sensitivities to commonly used drugs for HCC. The results showed that the high‐risk group was more sensitive to regorafenib, tivantinib, cabozantinib, cediranib, olaparib, navitoclax, mitomycin, vincristine, and paclitaxel, while the low‐risk group was more sensitive to erlotinib, brivanib, dasatinib, linifanib, neratinib, fluorouracil, and methotrexat (Figure 9). GSEA revealed that activated pathways in the high‐risk group were mainly associated with cancer, spliceosome, cell cycle, P53 signaling, and mismatch repair. The pathways activated in the low‐risk group involved more physiological functions of the liver (Figure 10).

## 4 DISCUSSION

Liver cancer remains a growing threat to global health. HCC accounts for approximately 90% of liver cancer cases and is the fastest rising cause of cancer mortality.^1^ Currently, TNM staging is the most widely used system for prognosis prediction that only considers histopathological factors. In addition, given the excessive heterogeneity of HCC, individual‐tailored treatment, and accurate risk stratification are critical to improving therapeutic efficacy and overall survival. Therefore, it is highly desirable to develop a panel of molecular markers to complement the TNM staging system.

Necroptosis is a regulated form of cell death that promotes inflammation and serves as an alternative defense mechanism when apoptosis is inhibited.^49^ As apoptosis escape is a well‐established feature of tumors, necroptosis is consequently initiated via the formation of necrosomes (the RIPK1–RIPK3–MLKL complex), which may exert a double‐edged sword effect on carcinogenesis.^14^ For example, downregulation of MLKL levels implies poor prognosis in ovarian and colon cancer, while increased expression levels of RIP1 confers a worse prognosis in glioblastoma.^9^, ^50^, ^51^ Paradoxically, one study found a protumor effect of RIPK1 in HCC patients, but most human hepatoma cell lines, including Huh‐7, HepG2, and Hep3B, suppress necroptosis by methylation‐dependent loss of RIPK3 expression.^14^, ^52^ Therefore, large‐scale and comprehensive analyses are warranted to investigate the roles of necroptosis in HCC patients.

Sample classification based on predefined gene sets is a widely applied method. We identified two necroptosis‐related clusters in the TCGA‐LIHC cohort and found that upregulation of the levels of these genes was related to decreased OS. To achieve better predictive ability, we developed a prognostic model from DEGs between the two molecular subgroups rather than merely using the necroptosis‐related genes. In the 10‐gene signature, two genes (S100A9 and MMP1) were directly regulated by the RIPK1‐dependent NF‐κB pathway, and the risk score was positively associated with the expression levels of most necroptosis‐related genes.^53^, ^54^, ^55^ It has been reported that necroptosis leads to inflammation, necrosis, fibrosis, and eventually oncogenesis in patients with viral hepatitis, alcoholic liver disease, and nonalcoholic fatty liver disease.^14^ Additionally, our results revealed that the presence of most immune cells and especially of protumor cells was positively correlated with the risk score. MDSCs, Th2 cells, and Treg cells can compromise normal immune surveillance and contribute to immune escape, while CAFs secrete growth factors and cytokines that favor tumor growth.^47^, ^56^ The influx of neutrophils mediated by interleukin‐17 produced by Th17 cells can drive tumor progression via the formation of neutrophil extracellular traps (NETs).^57^, ^58^ Collectively, we concluded that necroptosis was more likely to cause HCC progression than inhibition.

In the present study, the novel gene signature and the nomogram were developed based on the TCGA cohort and validated externally in the GEO and ICGC cohorts. The performance of the nomogram integrating the TNM stage and the 10‐gene signature was superior to that of the two independent prognostic predictors alone. Except for MTMR2, the other nine genes comprising the signature were previously reported in HCC and their functions could be roughly classified into five categories, including cell cycle (CDCA8), chronic inflammation (S100A9), anti‐oncogene (ANXA10), energy metabolism (G6PD, SLC1A5, SLC2A1), and extracellular matrix organization (SPP1, PLOD2, and MMP1). As a member of the myotubularin family of phosphoinositide lipid phosphatases, MTMR2 promotes gastric cancer development via the IFNγ/STAT1/IRF1 pathway and facilitates NK/T‐cell lymphoma progression by targeting JAK1.^59^, ^60^ The CDCA8 protein is a crucial regulator of mitosis and is overexpressed during hepatocarcinogenesis.^61^ S100A9 is a DAMP molecule that can amplify inflammation in the tumor microenvironment and lead to malignancy progression.^55^ G6PD encodes glucose‐6‐phosphate dehydrogenase, which limits the rate of the pentose phosphate pathway and can induce EMT in HCC cells through the STAT3 pathway.^62^ Increased glutamine metabolism is one of the critical metabolic modes of cancer cells.^63^ Upregulation of SLC1A5 levels is associated with increased glutamine uptake and dismal prognosis in hepatic cancer.^64^ Glucose transporter 1 (GLUT1) encoded by SLC2A1 is the primary regulator of glucose uptake and contributes to the metastasis and chemoresistance of liver cancer.^63^ Osteopontin (OPN) was first isolated from osteoblasts and is highly associated with bone mineralization.^65^ In liver tissues, OPN is encoded by SPP1 and correlates with the extent of liver regeneration, cirrhosis, and tumor growth.^66^ PLOD2 protein, also known as lysyl hydroxylase, is an independent prognostic factor of HCC and modulates collagen maturation.^67^, ^68^ In a hypoxic environment, increased PLOD2 expression levels promote the migration of pancreatic cancer cells by remodeling the extracellular matrix.^69^ Matrix metalloproteinase 1 (MMP‐1) degrades the pericellular matrix, leading to enhanced invasion and metastasis.^70^

Immunotherapy is one of the revolutions in oncology that ameliorates the conditions of patients diagnosed at unresectable stages. However, 60%–80% of patients are not responsive to ICI treatments in most cancer types.^71^ Thus, distinguishing between immune hot and immune cold tumors is crucial to improving prognosis.^72^ In the present study, the high‐risk group had significantly higher immune checkpoint activity (such as PD1 and CTLA4 activity), greater infiltration of antitumor cells (such as CD8+ T cells and B cells), and lower TIDE scores, indicating that high‐risk patients were immune hot. Conventionally, the roles of B cells in the fight against tumors are frequently neglected. However, B cells can assist in anti‐CTLA4 treatment by secreting antibodies.^73^ Thus, immunotherapy is more appropriate for the high‐risk group, which will reactivate the functionally suppressed CD8+ T cells and trigger antitumor immunity. Moreover, this gene signature could also predict responses to anti‐PD1/PD‐L1 treatments for patients with metastatic urothelial cancers in the IMvigor210 cohort. In contrast, the cold tumor environments of low‐risk patients should be converted to hot ones before the administration of immunotherapeutics, such as through phototherapy in combination with hypoxia‐activated chemotherapy.^74^ Chemotherapy is another effective strategy for advanced HCC. Because drug sensitivity is influenced by numerous genes instead of several markers, whole‐genome ridge regression has been introduced as a reliable forecasting method.^36^, ^75^ Based on the EC50 and transcription data of cell lines, we estimated the responses to commonly used chemotherapeutics for HCC. The comparison results revealed that the high‐risk patients were more resistant to erlotinib, brivanib, dasatinib, linifanib, neratinib, fluorouracil, and methotrexate. The chemoresistance might result from the activation of signaling pathways related to the spliceosome,^76^ mismatch repair,^77^ and cell cycle.^78^ However, several drugs were recommended for the high‐risk group, including regorafenib, tivantinib, cabozantinib, cediranib, olaparib, navitoclax, mitomycin, vincristine, and paclitaxel. Thus, the necroptosis‐related signature is conducive to treatment optimization. Moreover, with advancements in circulating tumor cell (CTC) detection and single‐cell sequencing, the treatment regimen can be dynamically adjusted during the treatment course.

Although we conducted comprehensive analyses and multidatabase validations, several limitations inherent to this study should be noted. First, LASSO penalization ignores some crucial genes regulating hepatocarcinogenesis. As the drug sensitivity predictions were based on bioinformatic analyses, large‐scale clinical trials are warranted to confirm our findings. Moreover, the mechanism of MTMR2 in HCC remains to be elucidated due to a lack of relevant research.

## 5 CONCLUSIONS

In summary, the novel necroptosis‐related gene signature is an independent prognostic factor of HCC and is reliable in prognosis prediction. In addition, it provides new evidence for individualized therapy. The nomogram can further improve predictive accuracy.

## AUTHORS' CONTRIBUTIONS

XDT and JLC conceived and planned this study. ZHL, JLC, HTW, ZL, and HC helped interpret the results. JLC took the lead in writing the manuscript. All authors provided vital feedback to shape the final version of the manuscript.

## CONFLICT OF INTEREST

The authors declare that they have no competing interests.

## Supporting information


Figure S1

Figure S2

Figure S3

Figure S4

Figure S5

Table S1

Table S2

Table S3

Table S4

Table S5

Table S6

## Data Availability

All data analyzed in the present study are publicly available in The Cancer Genome Atlas (TCGA; https://portal.gdc.cancer.gov/repository), International Cancer Genome Consortium (ICGC; https:/dcc.icgc.org/), Gene Expression Omnibus (GEO; https://www.ncbi.nlm.nih.gov/geo/), Human Protein Atlas (HPA; https://www.proteinatlas.org/), and SRING (http://string‐db.org) databases.
