# Supplementary figures and images for: A necroptosis‐related gene signature for predicting prognosis, immune landscape, and drug sensitivity in hepatocellular carcinoma
Source: Cancer Med. 2022 May 13;11(24):5079–96. doi: 10.1002/cam4.4812 (PMC9761093; doi:10.1002/cam4.4812)

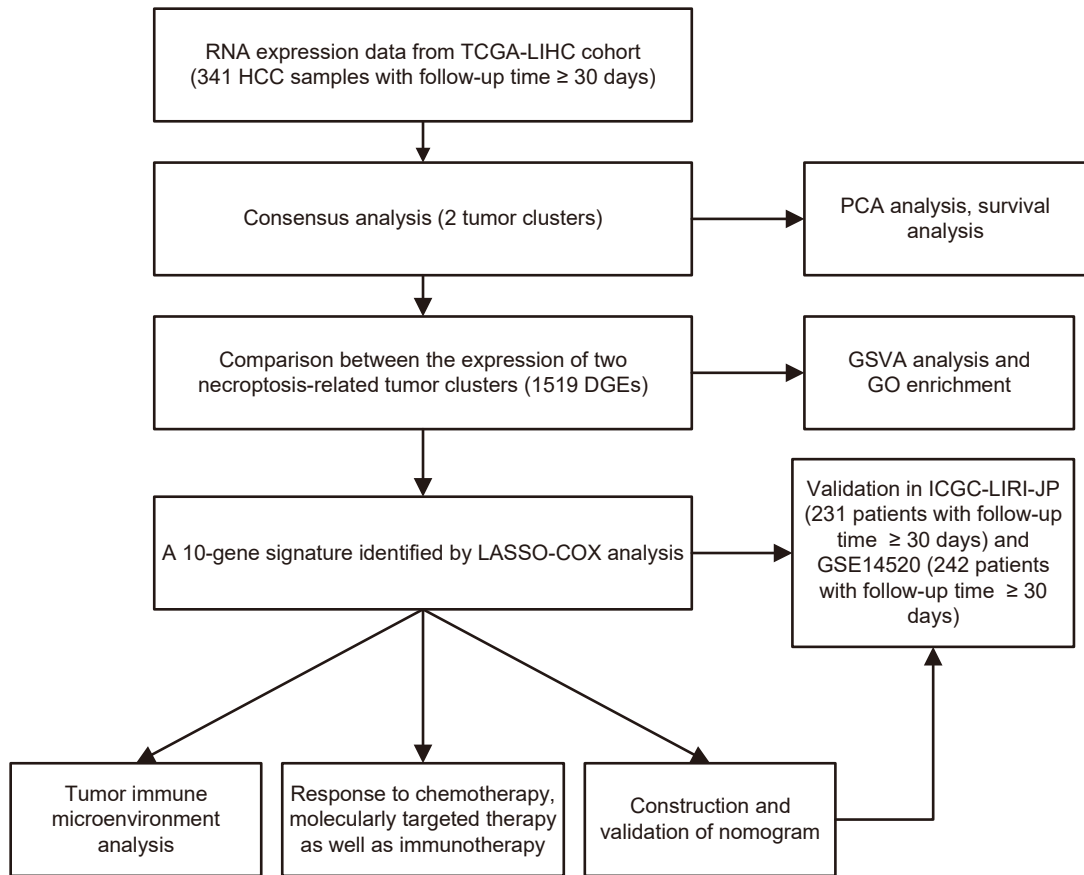

Supplement: Supplementary file 1 — Figure S1 [file CAM4-11-5079-s008.pdf]

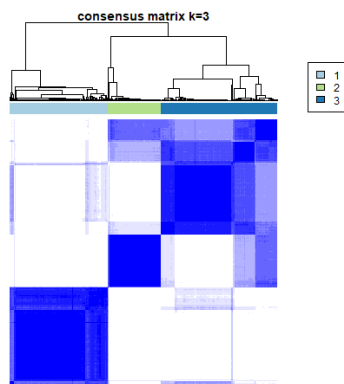

(A)

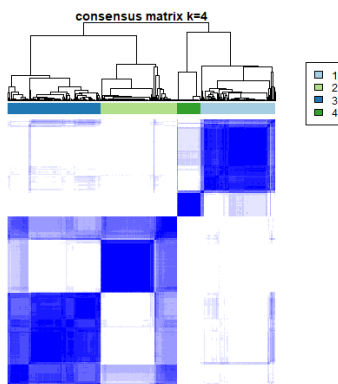

(B)

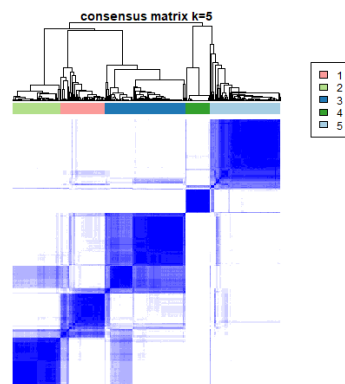

(C)

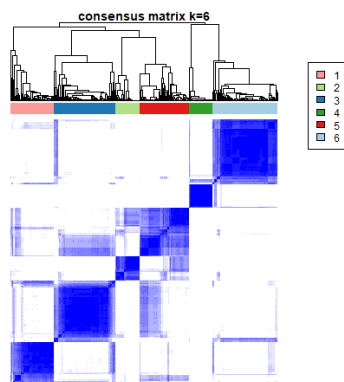

(D)

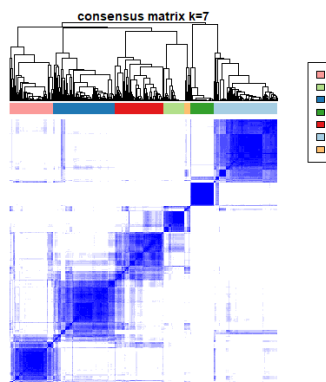

(E)

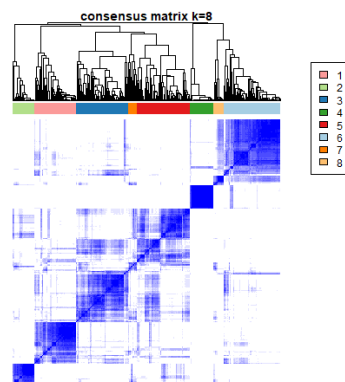

(F)

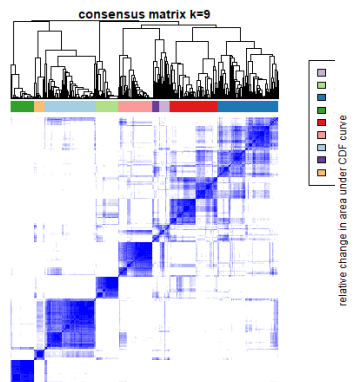

(G)

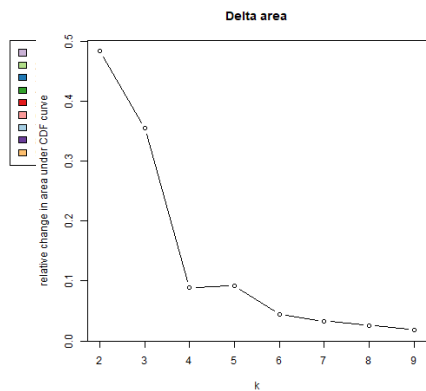

(H)

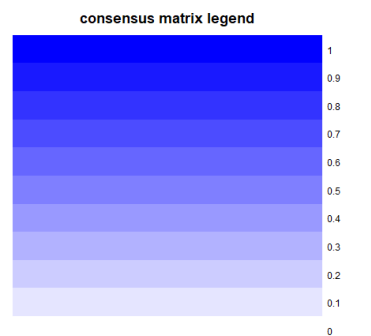

(I)

Supplement: Supplementary file 2 — Figure S2 [file CAM4-11-5079-s010.pdf]

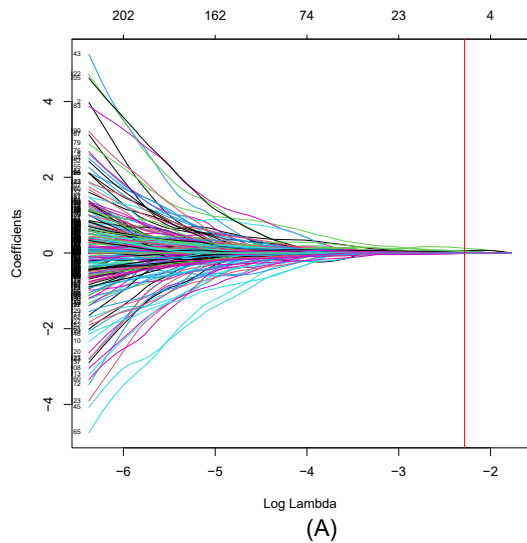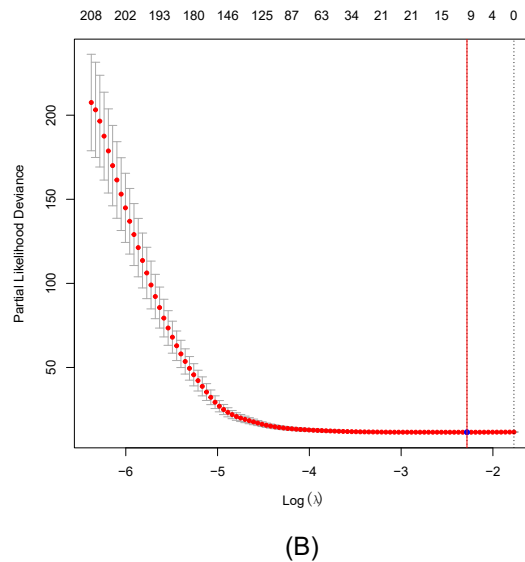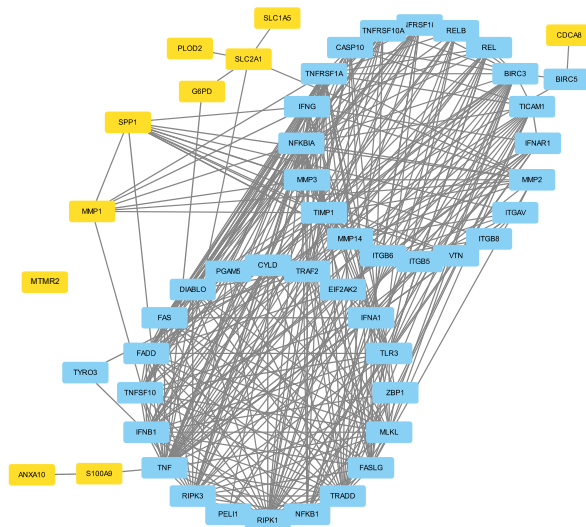

Supplement: Supplementary file 3 — Figure S3 [file CAM4-11-5079-s002.pdf]

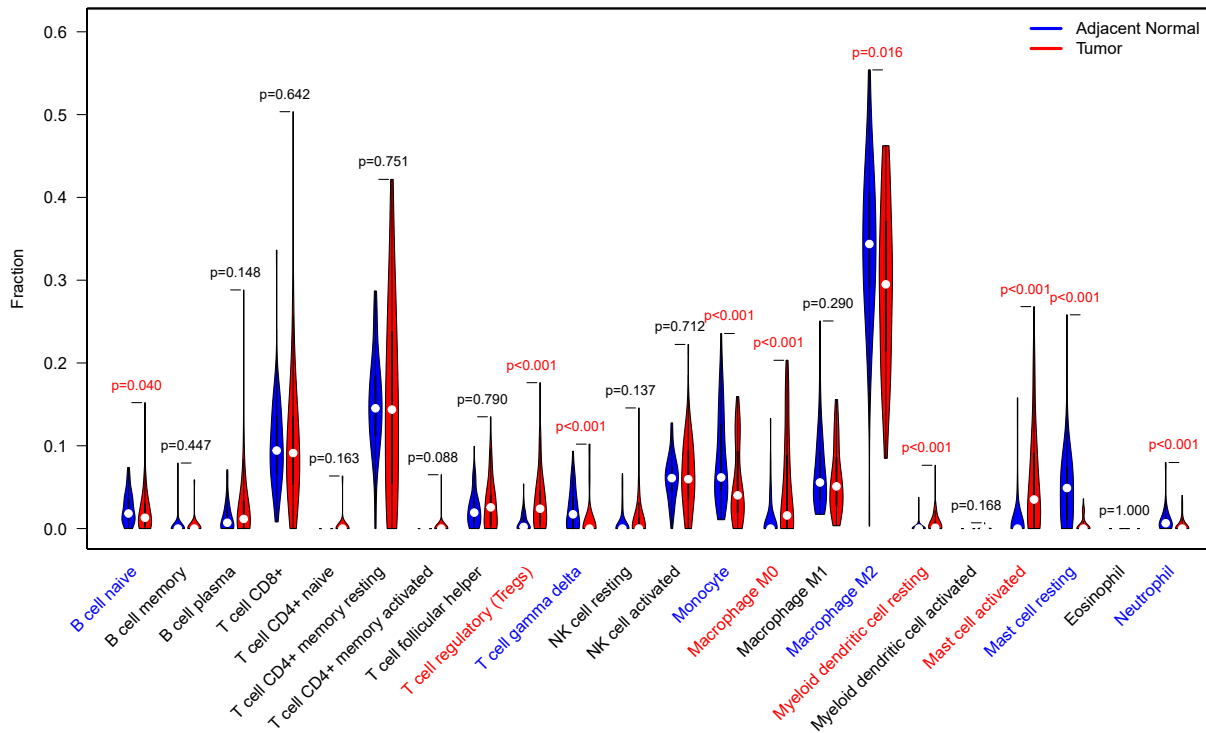

Supplement: Supplementary file 4 — Figure S4 [file CAM4-11-5079-s006.pdf]

Net Benefit

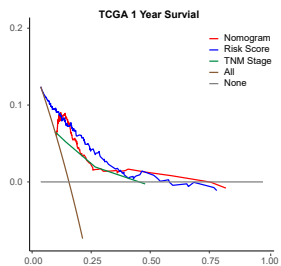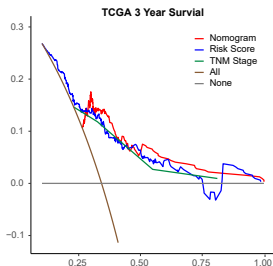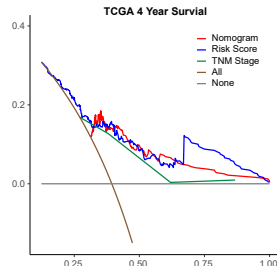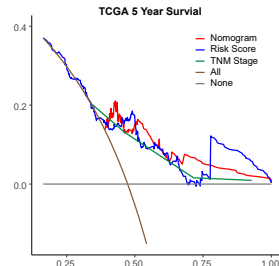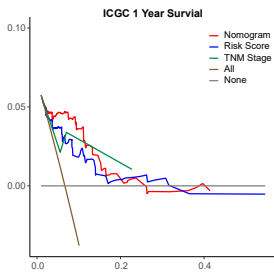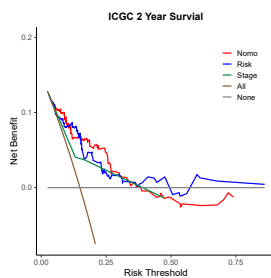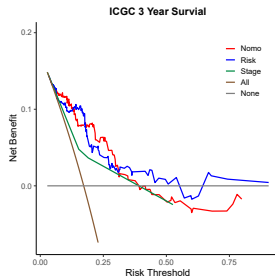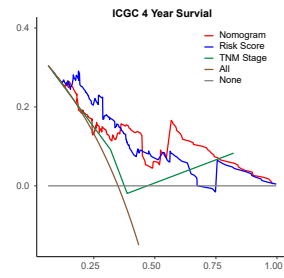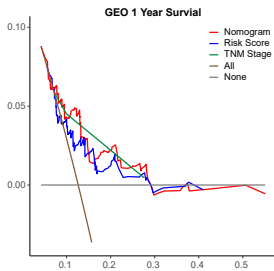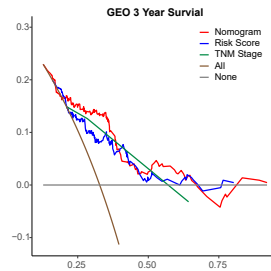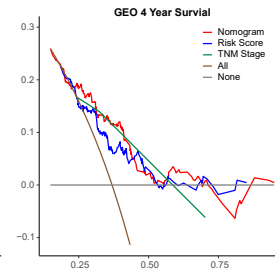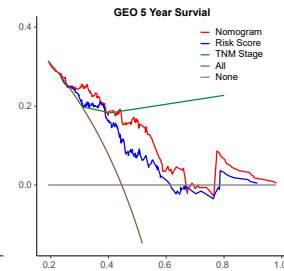

Risk Threshold

Supplement: Supplementary file 5 — Figure S5 [file CAM4-11-5079-s003.pdf]
